# Probiotic-facilitated cytokine-induced killer cells suppress peritoneal carcinomatosis and liver metastasis in colorectal cancer cells

**DOI:** 10.7150/ijbs.101051

**Published:** 2024-11-11

**Authors:** Hong-Hwa Chen, Chi-Wen Luo, Yi-Ling Chen, John Y. Chiang, Chi-Ruei Huang, Yi-Ting Wang, Chih-Hung Chen, Jun Guo, Hon-Kan Yip

**Affiliations:** 1 Division of Colorectal Surgery, Department of Surgery, Kaohsiung Chang Gung Memorial Hospital and Chang Gung University College of Medicine, Kaohsiung 83301, Taiwan.; 2 Division of Breast Oncology and Surgery, Department of Surgery, Kaohsiung Medical University Hospital, Kaohsiung Medical University, Kaohsiung City 807, Taiwan.; 3 Department of Cosmetic Science and Institute of Cosmetic Science, Chia Nan University of Pharmacy and Science, Tainan 717, Taiwan.; 4 Division of Cardiology, Department of Internal Medicine, Kaohsiung Chang Gung Memorial Hospital and Chang Gung University College of Medicine, Kaohsiung 83301, Taiwan.; 5 Institute for Translational Research in Biomedicine, Kaohsiung Chang Gung Memorial Hospital, Kaohsiung 83301, Taiwan.; 6 Department of Computer Science and Engineering, National Sun Yat-Sen University, Kaohsiung 80424, Taiwan.; 7 Center for Shockwave Medicine and Tissue Engineering, Kaohsiung Chang Gung Memorial Hospital Kaohsiung 833401, Taiwan.; 8 Divisions of General Medicine, Department of Internal Medicine, Kaohsiung Chang Gung Memorial Hospital and Chang Gung University College of Medicine, Kaohsiung 83301, Taiwan.; 9 Department of Cardiology, The First Affiliated Hospital, Jinan University, Guangzhou 510632, China.; 10 Department of Medical Research, China Medical University Hospital, China Medical University, Taichung 40402, Taiwan.

**Keywords:** colorectal cancer, tumor mass, liver metastasis, cell survival signaling, probiotics, cytokine-induced killer cell

## Abstract

**Background:** This study tested the hypothesis that combined therapy with probiotics and cytokine-induced killer (CIK) cells was superior to merely one on suppressing the peritoneal carcinomatosis and liver metastasis of colorectal cancer (CRC) cells in nude mice.

**Methods and Results:** The *in vitro* study revealed that in HCT 116/SW620 CRC cell lines, cell viability, proliferation, colony formation, migratory ability, wound healing, and protein expression of PD-L1 and FAK were significantly and comparably suppressed and that apoptosis was significantly and comparably increased by probiotics and CIK cells, and these effects were further significantly enhanced by combined probiotics + CIK cell therapy (all p<0.001). Nude mice were categorized into Groups 1 (SC), 2 (HCT 116), 3 (HCT 116 + probiotics), 4 (HCT 116 + CIK cells), and 5 (HCT 116 + probiotics + CIK cells). CRC cells were intraperitoneally implanted into Groups 2 to 5, and the animals were euthanized by Day 28. The results demonstrated that the abdominal dissemination of CRC cells, tumor numbers, tumor weights, liver weights, liver necrosis areas and the expression of γ-H2AX/PD-L1/FAK in harvested liver tumors were lowest in Group 1, highest in Group 2, and significantly and progressively decreased in Groups 3 to 5 (all p<0.0001). The protein expression levels of apoptotic and DNA damage biomarkers (Bax/c-caspase 3/c-PARP/γ-H2AX), a metastatic biomarker (FAK) and three tumor proliferation and survival signaling biomarkers (JAK-STAT1, PI3K/Akt/m-TOR and Ras/Raf/MEK/ERK) exhibited identical patterns to that of a tumor immune escape biomarker (PD-L1) among the groups (all p<0.0001).

**Conclusion:** The combination of probiotics and CIK cells was superior to either therapy alone in suppressing CRC cell growth, proliferation, liver metastasis and survival, mainly through downregulating cell proliferation and survival signaling pathways.

## Introduction

Colorectal cancer (CRC) is the third most commonly diagnosed malignancy worldwide, with 1.4 million new cases and 694,000 deaths globally 1, 2. The good news is that the mortality rate has slowly decreased by 2-3% annually 3 owing to improvements in treatment within these few years, especially in patients in the early stages of CRC. Undoubtedly, despite the notable progress achieved in the treatment of CRC, surgery remains the most suitable management method for patients, in contrast to chemotherapy, radiation and targeted therapy, suggesting that surgical intervention plays a pivotal role in CRC management 3-5. On the other hand, CRC metastasis remains the primary cause of cancer-related mortality and the decrease in the survival rate. Metastasis involves a multistep cellular process in which tumor cells disseminate from the primary tumor, invade through the basement membrane, travel through the circulatory system, and establish themselves in secondary tissues such as the liver or lungs 6.

Among patients with stage IV CRC, those with peritoneal carcinomatosis (PC) have the poorest prognostic outcome, with an estimated one-year survival rate of only 10% 4, even among those receiving different palliative treatments 5-7. Accordingly, an unacceptably high mortality rate and extremely low one-year survival rate increase the urgency of finding safe and relatively efficacious therapeutic options to improve the lives of patients with advanced CRC, which is highly important to physicians, especially for patients who are refractory to conventional therapy.

An increasing number of studies have shown that cancer immunotherapy is the 5^th^ most common cancer treatment modality behind surgery, chemotherapy, targeted therapy, and radiotherapy 8-11. In contrast to the other four therapies, cancer immunotherapy 12, 13 mainly focuses on enhancing the anticancer abilities of immune cells rather than directly killing cancer cells 14-16. One potential way to reconstitute host immunity is adoptive immunotherapy. In fact, adoptive immunotherapy can eliminate cancer cells through the transfusion of *ex vivo* expanded and activated immune cells, such as cytokine-induced killer (CIK) cells 17-20.

More than thirty-three years ago, Schmidt-Wolf *et al.* described a heterogeneous cell population, which included CD3 and CD56 antigens as the major components, that were activated and then rapidly expanded within peripheral blood mononuclear cells in the presence of interferon (IFN)-γ, an anti-CD3 monoclonal antibody and interleukin-2 (IL-2) stimulation *in vitro*. These cells subsequently differentiate into polyclonal T lymphocytes with a phenotype and function similar to those of NK cells 21 This heterogeneous population has since been designated as cytokine-induced killer (CIK) cells. Since then, autologous CIK cells from patients' peripheral blood mononuclear cells (PBMCs) have been activated and expanded *ex vivo* and subsequently transfused back into the patients 18, 22. CIK cells can undergo activation and expansion 200- to 1000-fold over 14-21 days of *ex vivo* culture, initiated by priming with CD3 antibodies and cytokine stimulation (specifically, culture in the presence of IL-2 to acquire the CD56 antigen) 20, 23. *Ex vivo*-expanded CIK cells are characterized as CD3+ CD56+ cells and exhibit potent cytotoxic activity against various tumor cell lines and tumors in animal models. Several clinical trials have demonstrated that combining CIK cell immunotherapy with chemotherapy provides promising benefits compared to chemotherapy alone for patients with advanced lung cancer 22-26.

Previous studies have clearly identified the gut microbiota as a complex community essential for maintaining a dynamic metabolic ecological balance 27. The gut microbiota plays crucial roles in immune system maturation by stimulating the innate immune system early in life, promoting the development of intestinal-related lymphoid tissue, and enhancing acquired immunity through local and systemic immune responses 28. Additionally, the gut microbiota contributes to the synthesis and metabolism of nutrients, hormones, and vitamins while assisting in the removal of drugs and toxins. Under normal conditions, the gut microbiota helps maintain a state of "low-grade physiological inflammation," which provides a rapid and effective defense mechanism against pathogens 29. However, under pathological conditions, the gut microbiota has been implicated in the development of behavioral and cognitive dysfunctions, inflammatory reactions, immune dysregulation, metabolic disorders, and neuropathic pain 30-38. Numerous investigations have also highlighted an association between the gut microbiota and the progression and stage of CRC, indicating a significant interaction between the host microbiota and CRC 39-45. These findings underscore the need for new therapeutic strategies that combine immunotherapy with personalized probiotic adjuvant treatments. On the basis of these considerations, it is rational to believe that probiotics could increase the efficacy of CIK cell therapy in eradicating CRC, potentially improving PC in nude mice.

## Materials and Methods

### Ethical issues

All animal procedures were approved by the Institutional Animal Care and Use Committee at Kaohsiung Chang Gung Memorial Hospital (approval no. 2022031102) and conducted in accordance with the Guide for the Care and Use of Laboratory Animals. The animals were housed in an AAALAC-accredited facility at the hospital and maintained at a controlled temperature of 24 °C with a 12-hour light/dark cycle.

### CIK cell preparation and culture

The procedure for CIK cell preparation and induction was reported in our previous study 46, 47. Specifically, 20 mL of human peripheral blood was collected from healthy subjects for CIK cell induction. This study received approval from the Chang Gung Memorial Hospital Research Ethics Committee Institutional Review Board (IRB), and all the experimental protocols adhered to the IRB-approved guidelines.

After informed consent was obtained, peripheral blood was collected from each healthy donor in heparin-containing tubes (BD Vacutainer). Peripheral blood mononuclear cells (PBMCs) were isolated from volunteers by Ficoll-Hypaque (GE Healthcare) density‒gradient centrifugation, washed three times, and cultured in 25 cm² flasks at a concentration of 2 × 10⁶ cells/ml in X-VIVO 15 medium (Lonza, 04-418Q) supplemented with human interferon gamma (IFN-γ) (#300-02, PeproTech). The following day, the medium was supplemented with an anti-CD3 monoclonal antibody (clone OKT3; 16-0037-85, eBioscience) and interleukin-2 (IL-2; #200-02, PeproTech). The cells were incubated in a humidified atmosphere with 5% CO₂ at 37 °C, and the medium was changed every 2-3 days. After at least 14 days of culture, the cells were counted with a LUNA automated cell counter (Logos Biosystems, Inc., Annandale, VA, USA), and the expression of various surface markers was analyzed via flow cytometry.

### CRC cell lines cocultured with probiotics

The procedure and protocol for the cell culture were based on the previous reports with minimal modification 48-50. In detail, HCT 116 and SW620 cells, two colorectal cancer (CRC) cell lines, were evenly seeded into 6-well plates at a density of 1 × 10⁵ cells per well. The cells were cultured at 37 °C in a 5% CO₂ incubator for 14 hours to allow for cell adhesion. The cells in the probiotic mixture (PM) group were cocultured with 2 mL of the probiotic solution (1 × 10⁸ CFU/2 mL) at 37 °C for 8 hours. The control cells received the same amount of RPMI-1640 culture medium supplemented with 10% fetal bovine serum (FBS). Following 8 hours of coculture, the cells were collected for subsequent experiments.

To evaluate the ability of probiotics to inhibit the activity of CRC cell lines, the cells were categorized into four groups: (1) A1 [HCT 116 cells (2 × 10⁵) + RPMI-1640 culture medium], (2) A2 [SW620 cells (2 × 10⁵) + RPMI-1640 culture medium], (3) B1 [HCT 116 cells (2 × 10⁵) + probiotics (1 × 10⁸ CFU/2 mL, i.e., 2 mL of the probiotic mixture)], and (4) B2 [SW620 cells (2 × 10⁵) + probiotics (1 × 10⁸ CFU/2 mL)]. The cells were then collected for various experiments, including the MTT assay, colony formation assay, wound-healing assay, migration and invasion assay, and Ki67 staining, to assess CRC cell proliferation.

### Rationale for the use of human probiotics in rodents

*Lactobacillus* species are generally regarded as safe probiotics with a long history of diverse applications. Additionally, our previous study 51 demonstrated that while the dominant microbiota differed between humans and rodents, the percentages of Lactobacillales species were similar, at 3.1% in humans and 2.8% in rats. Therefore, PROBIOTICS (PNT_BIO_-RAY^TM^ containing *L. paracasei*; Kao An Biomedical Co., Ltd., Kaohsiung, Taiwan) was selected for use in the studied rodents. The dosage and probiotics used in this study were based on our previous findings 51.

### CRC cell lines cocultured with probiotics and CIK cells

The CRC cell lines, HCT 116 and SW620 cells, were evenly seeded into 6-well plates at 1 × 10^5^ cells per well. To evaluate whether the combination of probiotics and CIK cells was superior to either treatment alone, the cells were grouped into the following groups: (1) C1 [HCT 116 cells (2 × 10^5^), (2) C2 [SW620 cells (2 × 10^5^)], (3) D1 [HCT 116 cells (2 × 10^5^) + probiotics (1×10^8^ CFU)/2 mL], (4) D2 [SW620 cells (2 × 10^5^) + probiotics (1×10^8^ CFU)/2 mL], (5) E1 [HCT 116 cells (2 × 10^5^) + CIK cells cocultured with various effector to target (E:T) ratios of 0:1, 1:1, 5:1, 10:1 and 20:1], (6) E2 [SW620 cells (2 × 10^5^) + CIK cells cocultured with the various effector to target (E:T) ratios of 0:1, 1:1, 5:1, 10:1 and 20:1], (7) F1 (HCT 116 cells + probiotics + CIK cells) and (8) F2 (SW620 cells + probiotics + CIK cells ), respectively. After cell culture, we collected the cells for individual experiments, including Western blot for cleaved caspase 3, cleaved PARP, FAK, γ-H2AX, cytochrome C, and PD-L1, MTT assays for assessment of cell viability, wound healing assays for assessment of cell growth, migration assay for assessment of cell invasion, and flow cytometry for assessment of early and late apoptosis and the expression of programmed death-ligand 1 (*PD*-*L1*).

### Animal grouping and strategic management

The pathogen-free, adult male BALB/c nude mice (n=40 in each group) (Charles River Technology, BioLASCO Taiwan Co., Ltd., Taiwan), aged 8-10 weeks, after the 7-day adaptation period, were randomly allocated into Group 1 [sham-operated control (SC), with intraperitoneal injection of 1.0 mL culture medium], Group 2 (HCT 116 cells only, with implantation of 5 × 10^5^ cells in 100 µL of culture medium into the peritoneum), Group 3 [HCT 116 cells (5 × 10^5^) + probiotics (15 mg/day, oral administration for 7 days prior to and up to Day 21 after CRC cell implantation into the peritoneum (i.e., a total of 28 days))], Group 4 [HCT 116 cells (5 × 10^5^) + CIK cells (5×10^6^ in 200 µL of culture medium for the first time on Day 14 by intraperitoneal injection, followed by intravenous injection on Day 17 after CRC cell implantation into the peritoneum) and Group 5 (i.e., HCT 116 cells + probiotics + CIK cells).

The number of CRC cells utilized in the present study was based on our recent report 48 with some modifications. In this animal study, each nude mouse in Groups 3 and 5 was first treated with probiotics [15 mg (i.e., 1.5 billion colony-forming units) (CFU)/day/mouse, orally QD for 7 days prior to and for 21 days after tumor cell implantation]. The probiotics utilized in the present study were *Lactobacillus* species, which are generally regarded as safe probiotics with a historical and broad application.

The animals in each group were euthanized on Days 14, 21 and 28 after the intraperitoneal injection of CRC cells into the abdominal cavity, and the data were collected for individual experiments.

### MTT assay and assessment of colony formation units

The procedures for these assays were described in our previous report 52. For the MTT assay, 5000 cells were first plated in a well of a 96-well plate. After the indicated time, the culture medium was removed, followed by addition of 200 µl of MTT reagent. After further incubation for 30 minutes, the reagent was removed, and the cells were solubilized with 100 µl of DMSO. A spectrophotometer was used to record the optical density at 595 nm.

For evaluation of the number of colony formation units, 2000 cells were first plated in a well of a 6-well plate. After the cell colonies were visible without a microscope, they were washed with PBS and fixed with pure methanol. After removing the methanol residue and rehydrating with deionized water, the cell colonies were stained with 10% Giemsa solution. When the cell colonies were stained blue, the Giemsa solution was removed, and the samples were washed with tap water until the background was clean. After air drying, a picture was taken with a scanner, and the number of cell colonies was counted.

### Transwell invasion assay

The procedure was described in our previous report 52. The cells were plated in medium containing 0.1% FBS in the upper Boyden chamber, which was coated with 100 μL of 10% Matrigel, while the lower chamber contained medium supplemented with 10% serum. After 48 hours of incubation, the cells on the apical side of each insert were removed, and the invading cells on the basal side of the membrane were fixed and stained. To quantify invasive cells, five fields of view were selected from the periphery and center of the chamber. The number of invaded cells was counted in these five randomly chosen fields under a microscope, and this process was repeated for three independent experiments.

### Transwell migration assay

The procedure and protocol were described in our previous report 52. The cells were first trypsinized, and then 5 × 10^4^ cells were added to Boyden chambers with an 8 µm pore size (Millipore, Billerica, MA, USA) containing medium supplemented with 0.5% FBS. The culture plates contained assay medium supplemented with 10% FBS. After 24 hours of incubation, the cells on the bottom of the filter were fixed with methanol and stained with Giemsa (Sigma Corp.). The number of migrated cells in each chamber was counted in five randomly chosen fields under a microscope. This process was repeated for three independent experiments.

### Wound healing assay

The procedure was described in our previous report 52. For the wound healing assay, 3.5 × 10^4^ cells were seeded into 12-well plates with linear spacer inserts. After overnight culture, the cell monolayer was created by removing the linear spacer inserts. The cells were either left untreated or treated with probiotics for 24 hours, and the migration distance was measured with ImageJ software (National Institutes of Health, Bethesda, MD, USA).

### Flow cytometric quantification of apoptotic cells

The procedure was described in our previous report 52. In this study, the cells were cultured with probiotics or CIK cells. Initially, the upper layer of the culture medium was collected, followed by trypsinization of the lower layer of cells for 15 minutes. The percentages of viable and apoptotic cells were assessed by flow cytometry by double staining with annexin V and propidium iodide (PI).

### Western blot analysis

The procedure was described in our previous studies 51, 52. In detail, equal amounts (50 µg) of protein extracts were loaded and separated by SDS‒PAGE with 8-12% acrylamide gradients. The separated proteins were transferred onto a polyvinylidene difluoride (PVDF) membrane (Amersham Biosciences). Nonspecific sites were blocked by incubating the membrane overnight in blocking buffer (5% nonfat dry milk in Tris-buffered saline containing 0.05% Tween 20).

The membranes were then incubated with the indicated primary antibodies [cleaved-caspase 3 (1:1000, Cell Signaling), cleaved-PARP (1:1000, Cell Signaling), γ-H2AX (1:1000, Cell Signaling), programmed death-ligand 1 (PD-L1) (1:2000, Proteintech), interferon gamma (IFN-γ) (1:2000, Abcam), mitochondrial Bax (1:1000, Abcam), phosphorylated (p)-JAK2 (1:1000, Cell Signaling), p-STAT3 (1:1000, Abcam), Ras (1:1000, Abcam), β-Raf (1:1000, Cell Signaling), p-focal adhesion kinase (FAK) (1:2000, Abcam), p-MEK1/2 (1:1000, Cell Signaling), p-ERK1/2 (1:5000, Millipore), p-PI3K (1:1000, Cell Signaling), PI3K (1:1000, Cell Signaling), p-Akt (1:2000, Cell Signaling), Akt (1:2000, Cell Signaling), p-mTOR (1:1000, Cell Signaling), mTOR (1:1000, Cell Signaling), actin (1:6000, Millipore), and COXIV (1:5000, Abcam)] for 1 hour at room temperature.

Horseradish peroxidase-conjugated anti-rabbit IgG (1:6000, Sigma) or horseradish peroxidase-conjugated anti-mouse IgG (1:6000, Sigma) was used as the secondary antibody, with a 1-hour incubation at room temperature. The membranes were rinsed eight times within an hour, and the immunoreactive bands were visualized with enhanced chemiluminescence (ECL; Amersham Biosciences) after exposure to Biomax L film (Kodak). For quantification, ECL signals were digitized with Labwork software (UVP).

### Immunohistochemical (IHC) and immunofluorescent (IF) staining

The procedures were described in our previous studies 51, 52. For IHC and IF staining, rehydrated paraffin sections were initially treated with 3% H_2_O_2_ for 30 minutes and then incubated with Immuno-Block reagent (BioSB, Santa Barbara, CA, USA) for another 30 minutes at room temperature. The sections were subsequently incubated with primary antibodies against specific targets: Ki67 (1:500, Abcam), cytochrome C (1:500, Santa Cruz), Hsp60 (1:1000, Abcam), FAK (1:800, Cell Signaling), PD-L1 (1:200, Cell Signaling), and γ-H2AX (1:500, Abcam). The control sections were incubated with irrelevant antibodies. Immunostaining was visualized with an enhanced DAB kit (Abcam).

### Statistical analysis

All the data are presented as the means ± SDs. Differences between two groups were assessed by Student's t test. Continuous variables among multiple groups were compared by ANOVA, followed by the Bonferroni correction for post hoc between-group comparisons. Repeated-measures ANOVA was employed to analyze cell variability at different time points. SAS statistical software for Windows version 8.2 (SAS Institute, Cary, NC, USA) was used for data analysis. A probability value <0.05 was considered statistically significant.

## Results

### Probiotic treatment significantly inhibited the viability, colony formation, wound healing, and migratory and invasive capacities of CRC cells

**Figure 1:**
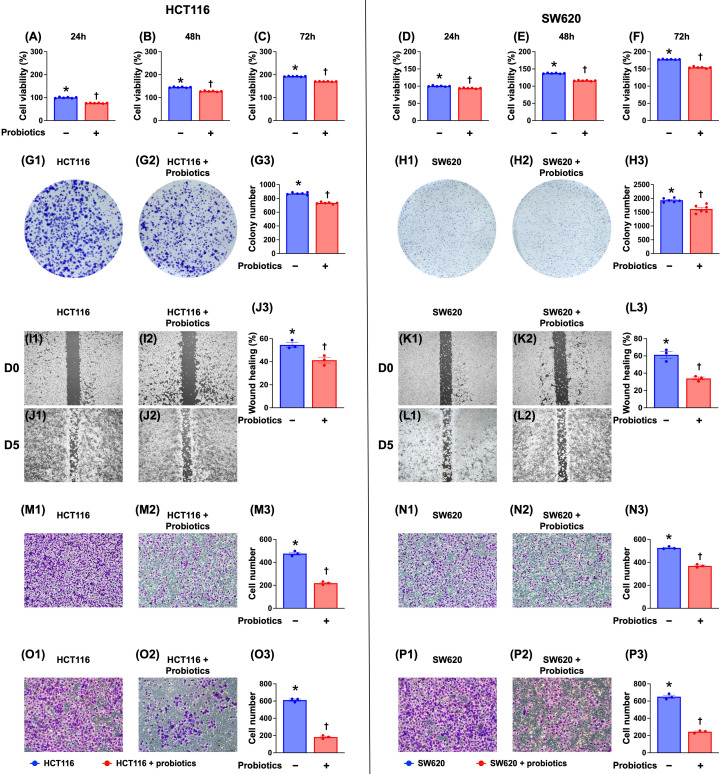
** Impact of probiotics treatment on suppressing the CRC cell viability, colony formation unit, wound healing process, and migratory and invasive capacities. A)** Cell viability of HCT 116 cells at 24 h, * vs. †, p <0.001 (n=6). **B)** Cell viability of HCT 116 cells at 48 h, * vs. †, p <0.001 (n=6). **C)** Cell viability of HCT 116 cells at 72 h, * vs. †, p <0.001 (n=6). **D)** Cell viability of SW620 cells at 24 h, * vs. †, p <0.001 (n=6).** E)** Cell viability of SW620 cells at 48 h, * vs. †, p <0.001 (n=6). **F)** Cell viability of SW620 cells at 48 h, * vs. †, p <0.001 (n=6). **G1 to G2)** Illustrating the colony formation unit (CFU) of HCT 116 cells (gray color). **G3)** Analytical result of number of CFU of HCT 116 cells, * vs. †, p <0.001 (n=6). **H1 to H2)** Illustrating the colony formation unit CFU of SW620 cells (gray color). **H3)** Analytical result of number of CFU of SW620 cells, * vs. †, p <0.001 (n=6). **I1 to I2)** Illustrating the baseline of wound healing process of HCT 116 cells that did not differ between two groups. **J1 to J2)** Illustrating the day 5 wound healing process of HCT 116 cells that was significantly suppressed by probiotics treatment. **J3)** Analytical result of wound healing process of HCT 116 cells at day 5, * vs. †, p <0.001 (n=6). **K1 to K2)** Illustrating the baseline of wound healing process of SW620 cells that did not differ between two groups. **L1 to L2)** Illustrating day 5 wound healing process of SW620 cells that was significantly suppressed in probiotics treatment. **L3)** Analytical result of wound healing process of SW620 cells at day 5, * vs. †, p <0.0001 (n=6). **M1 to M2)** Illustrating the microscopic finding (200x) for identification of migratory ability of HCT 116 cells (pink color). **M3)** Analytical result of number of migratory HCT 116 cells, * vs. †, p <0.0001 (n=6). **N1 to N2)** Illustrating the microscopic finding (200x) for identification of migratory ability of SW620 cells (pink color). **N3)** Analytical result of number of migratory SW620 cells, * vs. †, p <0.0001 (n=6). Scale bars in right lower corner represent 50µm. **O1 to O2)** Illustrating the microscopic finding (200x) for identification of invasive capacity of HCT 116 cells (pink color). **O3)** Analytical result of number of invasive HCT 116 cells, * vs. †, p <0.0001 (n=6). **P1 to P2)** Illustrating the microscopic finding (200x) for identification of invasive ability of SW620 cells (pink color). **P3)** Analytical result of number of invasive SW620 cells, * vs. †, p <0.0001 (n=6). Scale bars in right lower corner represent 50µm.

To determine whether probiotics are inhibitory to CRC cells, cell culture experiments were conducted. Our results (Fig. 1) indicated that probiotics significantly suppressed the viability, colony formation, wound healing, and migratory and invasive capacities of two CRC cell lines (HCT 116 and SW620 cells) compared with the control groups without probiotic treatments.

### The combination of probiotics and CIK cell therapy was superior to either treatment alone in suppressing the viability and migratory and invasive abilities of CRC cells

**Figure 2:**
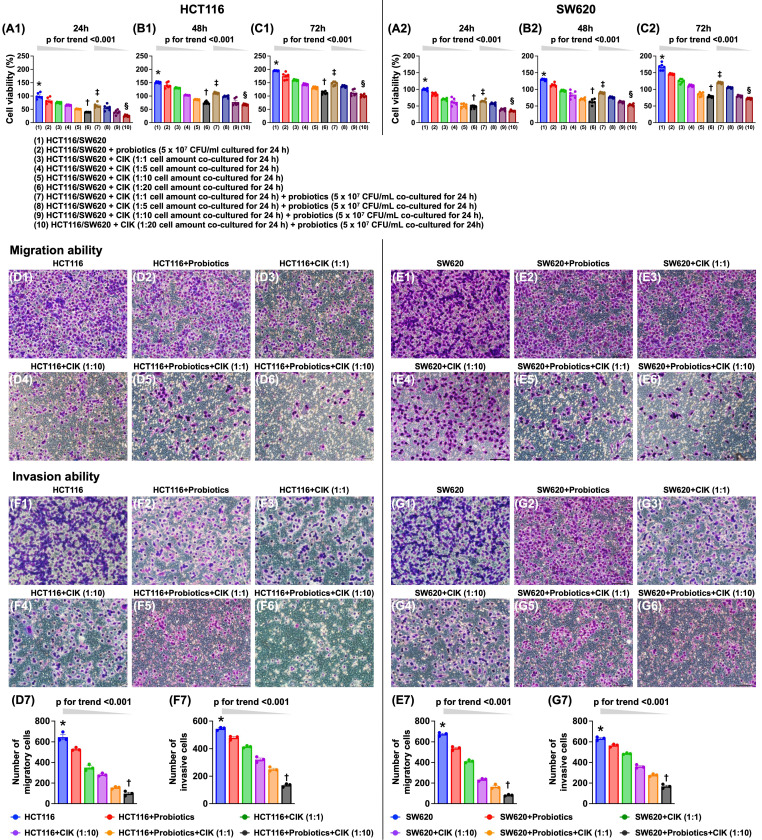
** Combined probiotics and CIK therapy was superior to merely either one on suppressing cell viability, migratory and invasive abilities of CRC cells. A1)** HCT 116 cell viability at 24 h, * vs. †, p<0.0001, p for trend <0.001; ‡ vs. §, p <0.0001, p for trend <0.001. **A2)** SW620 cell viability at 24 h, * vs. †, p<0.0001, p for trend <0.001; ‡ vs. §, p <0.0001, p for trend <0.001. **B1)** HCT 116 cell viability at 48 h, * vs. †, p<0.0001, p for trend <0.001; ‡ vs. §, p <0.0001, p for trend <0.001. **B2)** SW620 cell viability at 48 h, * vs. †, p<0.0001, p for trend <0.001; ‡ vs. §, p <0.0001, p for trend <0.001. **C1)** HCT 116 cell viability at 72 h, * vs. †, p<0.0001, p for trend <0.001; ‡ vs. §, p <0.0001, p for trend <0.001. **C2)** SW620 cell viability at 72 h, * vs. †, p<0.0001, p for trend <0.001; ‡ vs. §, p <0.0001, p for trend <0.001. **D1 to D6)** Illustrating the microscopic finding (200x) for identification of migration ability of HCT 116 cells (pink color). **D7)** Analytical result of number of migratory HCT 116 cells, * vs. †, p<0.0001; p for trend <0.001. **E1 to E6)** Illustrating the microscopic finding (200x) for identification of migration ability of SW620 cells (pink color). **E7)** Analytical result of number of migratory SW620 cells, * vs. †, p<0.0001; p for trend <0.001. **F1 to F6)** Illustrating the microscopic finding (200x) for identification of invasion ability of HCT 116 cells (pink color). **F7)** Analytical result of number of invasive HCT 116 cells, * vs. †, p<0.0001; p for trend <0.001. **G1 to G6)** Illustrating the microscopic finding (200x) for identification of invasion ability of SW620 cells (pink color). **G7)** Analytical result of number of invasive SW620 cells, * vs. †, p<0.0001; p for trend <0.001. Scale bars in right lower corner represent 50µm. CIK = cytokine-induced killer; CRC = colorectal cancer.

Compared with that of the control group, cell viability was significantly and progressively reduced by probiotic treatment and further decreased with increasing CIK cell concentrations (i.e., 1:1, 1:5, 1:10, and 1:20) at 24, 48, and 72 hours (Fig. 2 A1-C2). Additionally, when examining the cells subjected to the combined probiotic and CIK cell therapy, we found that cell viability also significantly and progressively decreased as the concentration of CIK cells increased (i.e., 1:1, 1:5, 1:10 and 1:20) at the same dose of probiotics (Fig. 2 A1-C2).

Additionally, the migratory assay demonstrated that, compared with that of the control group, the migratory ability of HCT 116 and SW620 cells was significantly and progressively suppressed by probiotic treatment alone and by stepwise increases in the concentration of CIK cells (i.e., 1:1 to 1:10), with the greatest suppression observed with the combined therapy of probiotics and stepwise increases in the concentration of CIK cells (i.e., 1:1 to 1:10). Furthermore, the invasive assay demonstrated that the invasive capacities of HCT 116 and SW620 cells exhibited an identical pattern as the migratory capacities with respect to the same conditions among the groups (Fig. 2 D1-G7).

### Effect of probiotics and CIK cells on the suppression of CRC proliferation, mitochondrial integrity and apoptosis

**Figure 3:**
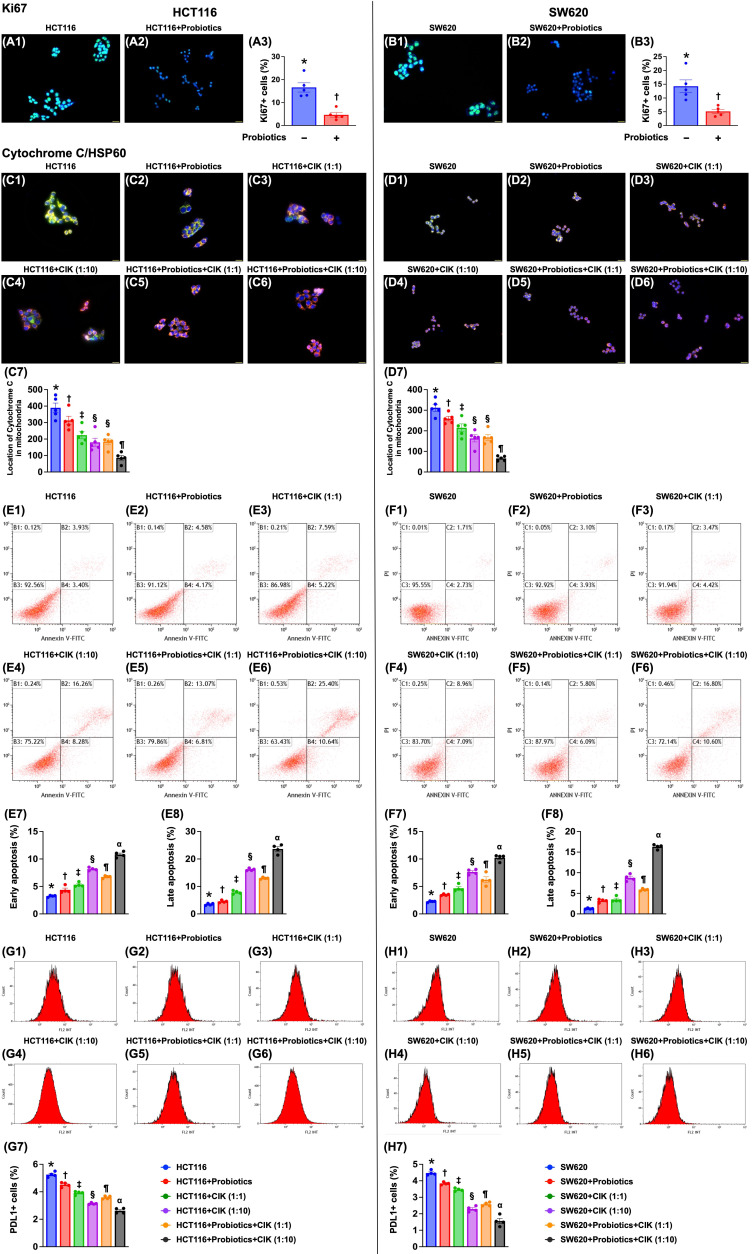
** Impact of probiotics and CIK on suppressing the CRC proliferation mitochondrial integrity. A1 to A2)** Illustrating the immunofluorescent (IF) microscopic finding (400x) for identification of ki67-positve stain of HCT 116 cells (green-blue color: indicated a merged picture of double stains of DAPI and ki67). **A3)** Analytical result of number of ki67+ cells, * vs. †, p<0.0001. **B1 to B2)** Illustrating the IF microscopic finding (400x) for identification of ki67-positve stain of SW620 cells (green-blue color: indicated a merged picture of double stains of DAPI and SW620). **B3)** Analytical result of number of ki67+ cells, * vs. †, p<0.0001. Scale bar in right lower corner represents 20µm. **C1 to C6)** Illustrating the immunofluorescent (IF) microscopic finding (400x) for identification of mitochondrial cytochrome C (green-red color) in HCT 116 [noted they were merged pictures of double stains by cytochrome C stain (green color) + heat-shock protein 60 (Hsp60) for identification of mitochondria (red color)]. **C7)** Analytical result of number of cytochrome C in mitochondria, * vs. other groups with different symbols (†, ‡, §, ¶) p<0.0001. **D1 to D6)** Illustrating the IF microscopic finding (400x) for identification of mitochondrial cytochrome C (green-red color) in SW620 [noted they were merged pictures of double stains by cytochrome C stain (green color) + heat-shock protein 60 (Hsp60) for identification of mitochondria (red color)]. **D7)** Analytical result of number of cytochrome C in mitochondria, * vs. other groups with different symbols (†, ‡, §, ¶) p<0.0001. Scale bars in right lower corner represent 20µm. **E1 to E6)** Illustrating the flow cytometric analysis for identification of early and late apoptoses of HCT 116 cells undergoing probiotics and CIK treatment. **E7)** Analytical result of number of early (AN-V+/PI-) apoptosis of HCT 116, * vs. other groups with different symbols (†, ‡, §, ¶, α) p<0.0001. **E8)** Analytical result of number of late (AN-V+/PI+) apoptosis of HCT 116, * vs. other groups with different symbols (†, ‡, §, ¶, α) p<0.0001. **F1 to F6)** Illustrating the flow cytometric analysis for identification of early and late apoptoses of SW620 cells undergoing probiotics and CIK treatment. **F7)** Analytical result of number of early (AN-V+/PI-) apoptosis of SW620, * vs. other groups with different symbols (†, ‡, §, ¶, α) p<0.0001. **F8)** Analytical result of number of late (AN-V+/PI+) apoptosis of SW620, * vs. other groups with different symbols (†, ‡, §, ¶, α) p<0.0001. **G1 to G6)** Illustrating the flow cytometric analysis for identification of PDL1 expression in HCT 116 cells, undergoing probiotics and CIK treatments. **G7)** Analytical result of number of PDL1-positive stain in HCT 116 cells, * vs. other groups with different symbols (†, ‡, §, ¶, α) p<0.0001. **H1 to H6)** Illustrating the flow cytometric analysis for identification of PDL1 expression in SW620 cells, undergoing probiotics and CIK treatments. **H7)** Analytical result of number of PDL1-positive stain in SW620 cells, * vs. other groups with different symbols (†, ‡, §, ¶, α) p<0.0001. All statistical analyses were performed by one-way ANOVA, followed by Bonferroni multiple comparison post hoc test (n=4-5 for each group). Symbols (*, †, ‡, §, ¶, α) indicate significance (at 0.05 level). CIK = cytokine-induced killer; CRC = colorectal cancer.

To assess whether probiotics and CIK cell treatment suppress cell proliferation and the number of mitochondria in CRC cells, immunofluorescence staining was conducted after cell culture. Compared with that in the control groups, the expression of Ki67, an indicator of cell proliferation, was significantly lower in HCT 116 and SW620 CRC cells treated with probiotics (Fig. 3 A1-B3).

Compared with that in the control groups, the expression of mitochondrial cytochrome C, an indicator of mitochondrial integrity, was significantly lower in probiotic-treated cells (Fig. 3 C1-D7). This reduction was even more significant in cells treated with low-dose CIK cells (1:1), further reduced in cells treated with high-dose CIK cells (1:10) or combined probiotics and low-dose CIK cells (1:1), and most pronounced in cells treated with combined probiotics and high-dose CIK cells (1:10) (Fig. 3 C1-D7). On the other hand, the percentage of HCT 116 and SW620 CRC cells in early and late apoptosis (Fig. 3. E1-F8) displayed an opposite pattern, whereas the expression of PD-L1 (Fig. 3 G1-H7) displayed a similar pattern to that of mitochondrial cytochrome C among the groups.

### Effect of probiotics and CIK cell therapies on the suppression of the protein expression of CRC immune escape-, migration- and death-related biomarkers

**Figure 4:**
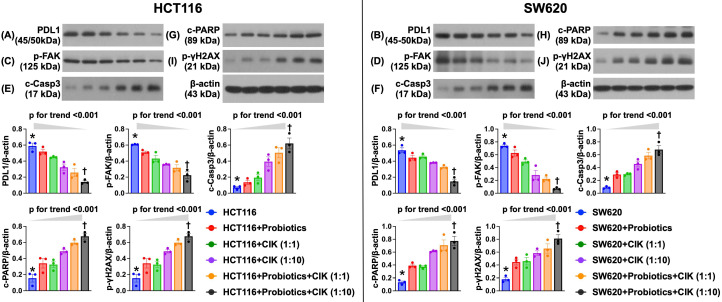
** Impact of probiotics and CIK therapies on suppressing the protein expression of CRC immune escape and migration-required biomarker, and CRC dead-related biomarkers. A)** Protein expression of programmed death protein 1 ligand (PD-L1) in HCT 116 cells, * vs. †, p< 0.0001; p for trend <0.001. **B)** Protein expression of PD-L1 in SW620 cells, * vs. †, p< 0.0001; p for trend <0.001. **C)** Protein expression of focal adhesion kinase (FAK) in HCT 116 cells, * vs. †, p< 0.0001; p for trend <0.001. **D)** Protein expression of FAK in SW620 cells, * vs. †, p< 0.0001; p for trend <0.001. **E)** Protein expression of cleaved caspase 3 (c-Casp3), in HCT 116 cells, * vs. †, p< 0.0001; p for trend <0.001. **F)** Protein expression of c-Casp3, in SW620 cells, * vs. †, p< 0.0001; p for trend <0.001. **G)** Protein expression of c-PARP in HCT116 cells, * vs. †, p< 0.0001; p for trend <0.001. **H)** Protein expression of c-PARP in SW620 cells, * vs. †, p< 0.0001; p for trend <0.001. **I)** Protein expression of γ-H2AX in HCR 116 cells, * vs. †, p< 0.0001; p for trend <0.001. **J)** Protein expression of γ-H2AX in SW620 cells, * vs. †, p< 0.0001; p for trend <0.001. n=6 for each group. CIK = cytokine-induced killer; CRC = colorectal cancer.

To elucidate the impact of probiotics and CIK cell therapies on regulating the protein levels of CRC immune escape- and migration-related biomarkers in HCT 116 and SW620 cells, Western blot analysis of an *in vitro* experiment was conducted. Compared with those in the control groups (i.e., HCT 116 and SW620 cells), the protein expression of *PD*-*L1*, an indicator of immune escape (i.e., adaptive immune resistance), and of focal adhesion kinase (FAK), an integrin-associated protein tyrosine kinase playing an essential role in transmitting signals for mediating several functions, including tumor cell proliferation, migration, adhesion and survival, were significantly suppressed by probiotic treatment in both HCT 116 and SW620 cells (Fig. 4 A-D). Additionally, these two parameters were further significantly suppressed by CIK cell treatment, especially at higher CIK cell concentrations (i.e., 1:1 vs. 1:10, p<0.001) (Fig. 4 A-D). Interestingly, the combined probiotics and CIK cell treatment further suppressed PD-L1 and FAK in HCT 116 and SW620 cells, especially when the concentration of CIK cells was increased (i.e., 1:1 vs. 1:10, p<0.001) (Fig. 4 A-D).

Western blot analysis revealed that the protein levels of cleaved caspase 3 and cleaved PARP, two apoptotic biomarkers, and the protein expression of γ-H2AX, an indicator of DNA damage in both HCT 116 and SW620 cells, displayed a pattern opposite pattern to that of PD-L1 among the groups (Fig. 4 E-J). Our findings highlighted that the combination of probiotics and CIK cells was superior to either treatment alone in suppressing CRC cell biological activity and enhancing the success of killing CRC cells.

### Time courses of the bioluminescence signal of CRC in the liver and abdomen and anatomical and pathological features of the tumors

CRC cells (Groups 2 (HCT 116 cells only), 3 (HCT 116 cells + probiotics), 4 (HCT 116 cells + CIK) and 5 (HCT 116 cells + probiotics + CIK cells)) were implanted into the peritonea of mice, with Group 1 serving as the SC.

**Figure 5:**
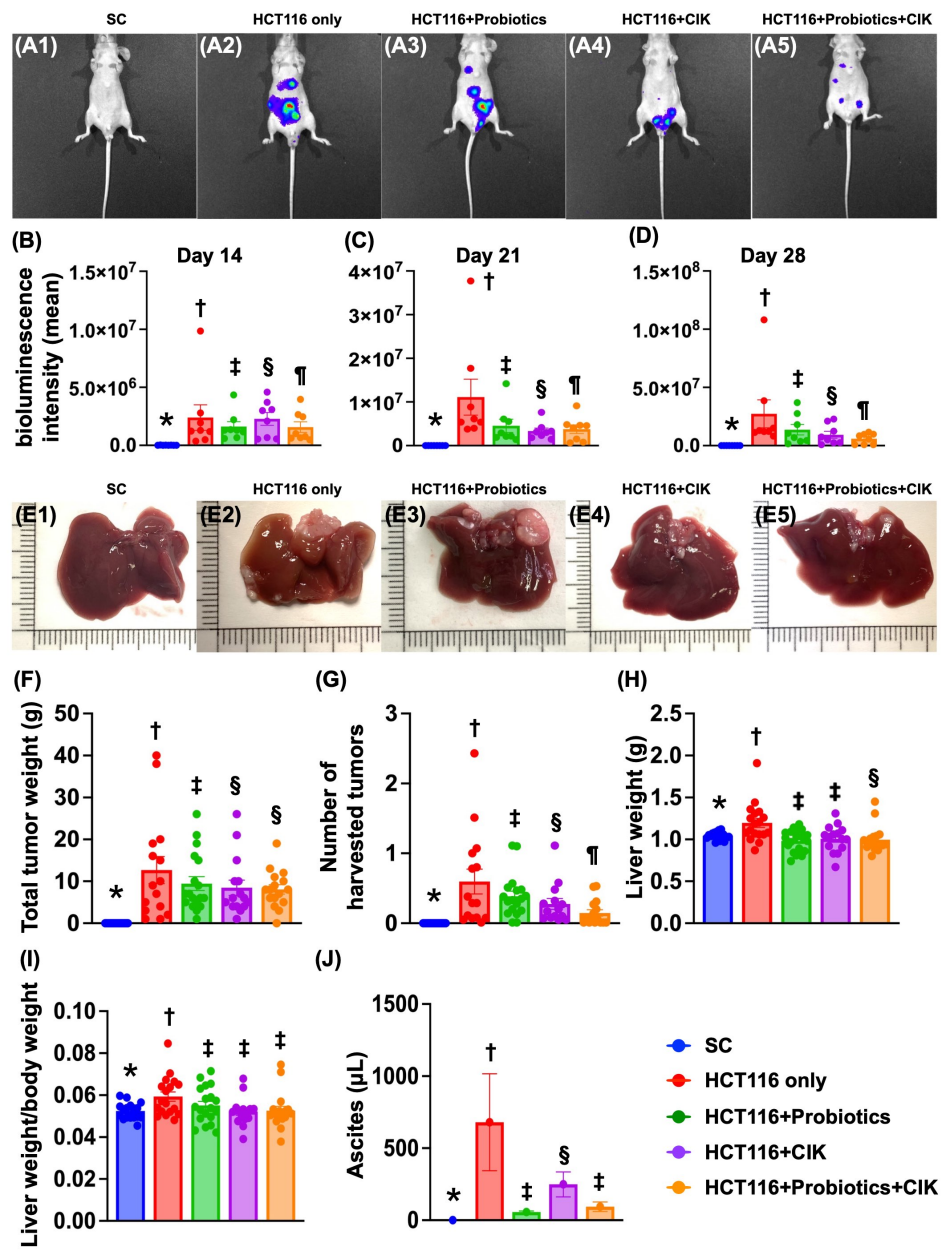
** Time courses of bioluminescence single of CRC in liver and abdomen, and anatomical and pathological features of tumor. A1 to A5)** Illustrating the day-28 IVIS finding for identification of bioluminescence signal intensity from implanted CRC cells (green fluorescence) in each group. **B)** Analytical result of bioluminescence signal intensity by day 14, * vs. other groups with different symbols (†, ‡, §, ¶) p<0.0001. **C)** Analytical result of bioluminescence signal intensity by day 21, * vs. other groups with different symbols (†, ‡, §, ¶) p<0.0001. **D)** Analytical result of bioluminescence signal intensity by day 28, * vs. other groups with different symbols (†, ‡, §, ¶) p<0.0001. For A to D, n=8 for each group. **E1 to F5)** Illustrating the morphological features of liver and the different tumor sizes harvested from liver of different groups. **F)** Total tumor weight, * vs. other groups with different symbols (†, ‡, §) p<0.0001. **G)** Analytical result of total number of harvested tumors in each group, * vs. other groups with different symbols (†, ‡, §, ¶) p<0.0001. **H)** liver weight, * vs. other groups with different symbols (†, ‡, §) p<0.0001. **I)** The ratio of liver weight to body weight, * vs. other groups with different symbols (†, ‡) p<0.001. For E to I, n=17-20 for each group. **J)** Total amount of ascites by day 28, * vs. other groups with different symbols (†, ‡, §) p<0.0001. n=8 for each group. All statistical analyses were performed by one-way ANOVA, followed by Bonferroni multiple comparison post hoc test. Symbols (*, †, ‡, §, ¶, α) indicate significance (at 0.05 level). group 1 = SC, group 2 = HCT 116 cells only, group 3 = HCT 116 cells + probiotics, group 4 = HCT 116 cells + CIK, group 5 = HCT 116 cells + probiotics + CIK. CIK = cytokine-induced killer; CRC = colorectal cancer.

First, when the IVIS was used to track the destination and bioluminescence signals of CRC cells, we found that the intensity of the bioluminescence signal on Day 14 after the implantation of CRC cells into the peritoneum was lowest in Group 1, highest in Group 2, significantly greater in Group 4 than in Groups 3 and 5 and significantly greater in Group 3 than in Group 5 (Fig. 5 A1-B). Additionally, by Day 21, this parameter exhibited a similar pattern as that on Day 14 among the groups (Fig. 5 A1-C). On the other hand, on Day 28 after CRC tumor induction, this parameter was lowest in Group 1, highest in Group 2 and significantly and progressively decreased from Groups 3 to 5 (Fig. 5 A1-D). Interestingly, the final destinations of the CRC cells were identified by IVIS, which revealed a diffuse dissemination in the subdiaphragm area, liver, and spleen and a scattering in the peritoneum (Fig. 5 A1-A5). Additionally, the pathological findings demonstrated that the tumors could be harvested from the liver subdiaphragmatic region and peritoneal cavity (Fig. 5 E1-E5, 5G).

Next, we carefully measured the amount of ascites in each group of animals and found that the volume of ascites was highest in Group 2, lowest in Group 1 and significantly greater in Group 4 than in Groups 3 and 5; however, there was no difference between these latter two groups (Fig. 5J).

We then carefully measured the total tumor weight and found that this parameter was significantly greater in Group 2 than in Groups 3 to 5 and significantly greater in Group 3 than in Groups 4 and 5, but there was no difference between the latter two groups (Fig. 5F). Additionally, when we carefully estimated the total number of tumors, we found that the tumors, with variable sizes and diameters, were significantly and progressively reduced from Groups 2 to 5 (Fig. 5G). Furthermore, the liver weights (Fig. 5H) and the ratios of liver weight to body weight (Fig. 5I) were significantly greater in Group 2 than in the other groups.

### Effect of probiotics and CIK cells on the expression of PD-L1 and FAK in liver tumors by Day 28 after tumor induction

**Figure 6:**
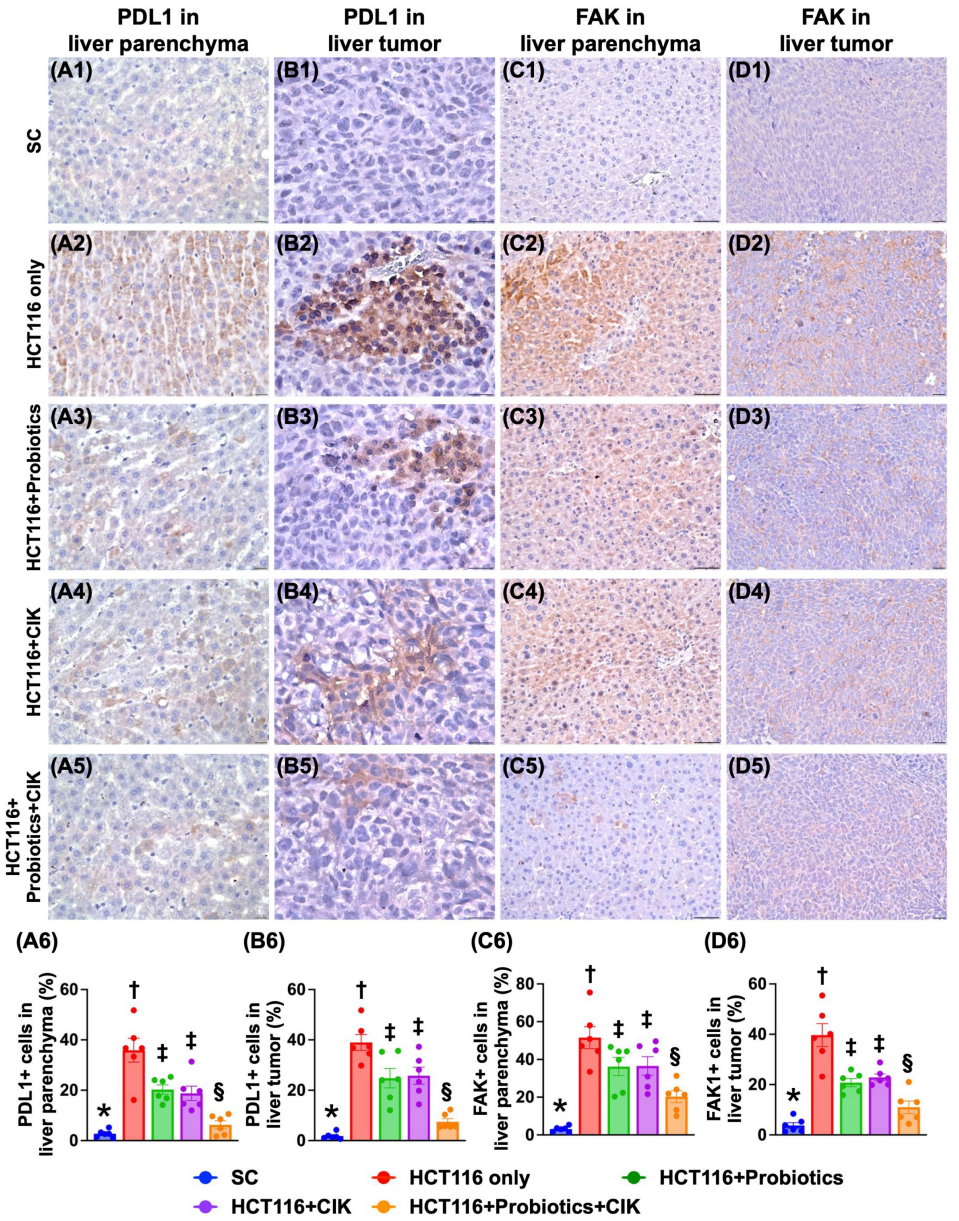
** Impact of probiotics and CIK on cellular expressions of PD-L1 and FAK in tumor and liver parenchyma by day 28 after CRC induction. A1 to A5)** Illustrating the microscopic finding (400x) of immunohistochemical (IHC) stain for identification of cellular expression of PD-L1 (gray) in liver parenchyma. Scale bar in right lower corner represents 20µm. **A6)** Analytical result of percentage (%) of PD-L1+ cells in liver parenchyma, * vs. other groups with different symbols (†, ‡, §) p<0.0001. **B1 to B5)** Illustrating the microscopic finding (800x) of IHC stain for identification of cellular expression of PD-L1 (gray) in liver tumor specimen. Scale bar in right lower corner represents 25µm. **B6)** Analytical result of percentage (%) of PD-L1+ cells in liver tumor specimen, * vs. other groups with different symbols (†, ‡, §) p<0.0001. **C1 to C5)** Illustrating the microscopic finding (320x) of IHC stain for identification of cellular expression of focal adhesion kinase (FAK) (gray) in liver parenchyma. Scale bar in right lower corner represents 50µm. **C6)** Analytical result of percentage (%) of FAK+ cells in liver tumor specimen, * vs. other groups with different symbols (†, ‡, §) p<0.0001. **D1 to D5)** Illustrating the microscopic finding (200x) of IHC stain for identification of cellular expression of FAK (gray) in liver tumor specimen. Scale bar in right lower corner represents 50µm. **D6)** Analytical result of percentage (%) of FAK+ cells in liver tumor specimen, * vs. other groups with different symbols (†, ‡, §) p<0.0001. All statistical analyses were performed by one-way ANOVA, followed by Bonferroni multiple comparison post hoc test (n=6 for each group). Symbols (*, †, ‡, §) indicate significance (at 0.05 level). CIK = cytokine-induced killer; CRC = colorectal cancer.

To assess the effect of probiotics and CIK cells on suppressing the levels of PD-L1 and FAK in liver tumors, IHC staining was utilized. The results revealed that the expression levels of PD-L1 and FAK in tumor and liver tissues were lowest in Group 1, highest in Group 2 and significantly lower in Group 5 than in Groups 3 and 4, but they did not differ between Groups 3 and 4 (Fig. 6 A1-D6).

### Liver necrosis area and expression of DNA damage biomarkers by Day 28 after tumor induction

**Figure 7:**
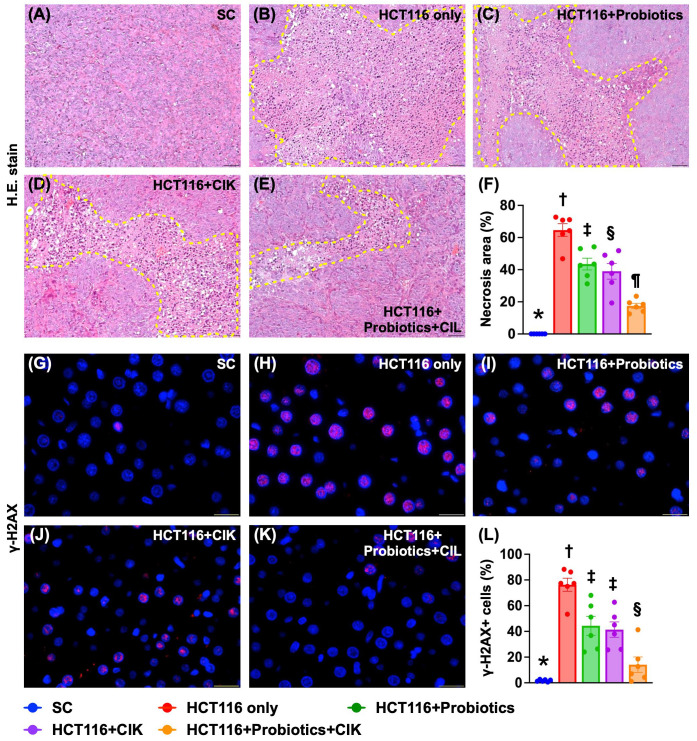
**Liver necrosis area and cellular expression of DNA damaged marker by day 28 after CRC induction. A to E)** Showing the H.E. stain (200x) for identification of liver necrosis (yellow-dotted line). Scale bar in right lower corner represents 50µm. **F)** Analytical result of necrosis area of liver, * vs. other groups with different symbols (†, ‡, §, ¶) p<0.0001. **G to K)** Illustrating the immunofluorescent microscopic finding (800x) for identification of cellular expression of γ-H2AX (red color), * vs. other groups with different symbols (†, ‡, §) p<0.0001. Scale bar in right lower corner represents 25µm. **L)** Analytical result of number of γ-H2AX+ cells, * vs. other groups with different symbols (†, ‡, §) p<0.0001. All statistical analyses were performed by one-way ANOVA, followed by Bonferroni multiple comparison post hoc test (n=6 for each group). Symbols (*, †, ‡, §) indicate significance (at 0.05 level). CRC = colorectal cancer.

H&E staining revealed that the liver necrosis area was lowest in Group 1, highest in Group 2, and significantly lower in Group 5 than in Groups 3 and 4, but this parameter was similar between these latter two groups (Fig. 7 A-F). Additionally, the IF staining microscopic findings demonstrated that the expression of γ-H2AX, an indicator of DNA damage, displayed an identical pattern to that of liver necrosis among the groups (Fig. 7 G-L).

### Effect of probiotics and CIK cells on regulating the protein expression of apoptosis and DNA damage biomarkers, PD-L1, and FAK in liver tumors by Day 28 after CRC tumor induction

**Figure 8:**
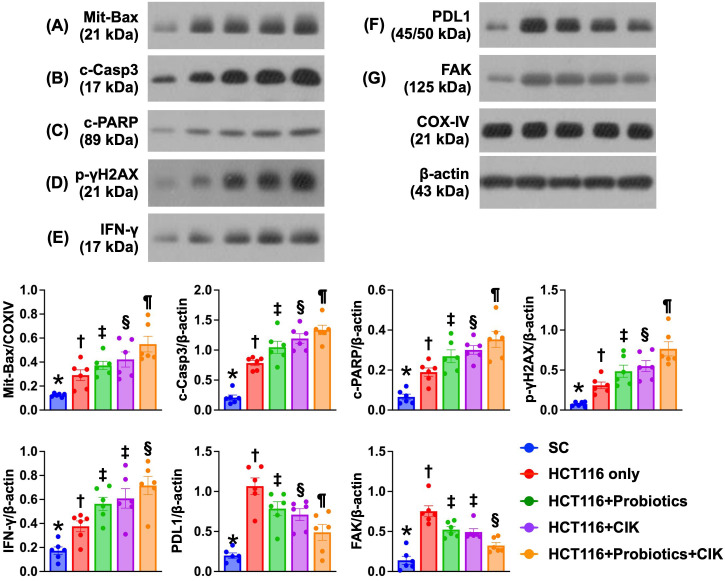
** Impact of probiotics and CIK on regulating the protein expressions of apoptosis and DNA-damaged, PD-L1 and FAK biomarkers in liver organ by day 28 after CRC tumor induction. A)** Protein expression of mitochondrial Bax (mit-Bax), * vs. other groups with different symbols (†, ‡, §, ¶) p<0.0001. **B)** Protein expression of cleaved caspase 3 (c-Casp3), * vs. other groups with different symbols (†, ‡, §, ¶) p<0.0001. **C)** Protein expression of cleaved PARP (c-PARP), * vs. other groups with different symbols (†, ‡, §, ¶) p<0.0001. **D)** Protein expression of γ-H2AX, * vs. other groups with different symbols (†, ‡, §, ¶) p<0.0001. **E)** Protein expression of interferon gamma (IFN-γ), * vs. other groups with different symbols (†, ‡, §) p<0.0001. **F)** Protein expression of programmed death-ligand 1 (*PD*-*L1*), * vs. other groups with different symbols (†, ‡, §, ¶) p<0.0001. **G)** Protein expression of focal adhesion kinase (FAK), * vs. other groups with different symbols (†, ‡, §) p<0.0001. n=6 for each group. All statistical analyses were performed by one-way ANOVA, followed by Bonferroni multiple comparison post hoc test (n=6 for each group). Symbols (*, †, ‡, §, ¶) indicate significance (at 0.05 level). CIK = cytokine-induced killer; CRC = colorectal cancer.

The protein expression of Bax, cleaved caspase 3 and cleaved PARP, three indices of apoptosis, and of γ-H2AX, an indicator of DNA damage, significantly and progressively increased from Groups 1 to 5 (Fig. 8 A-E). On the other hand, the protein expression levels of PD-L1 and FAK were lowest in Group 1 and significantly and progressively decreased from Groups 2 to 5 (Fig. 8 F-G).

### Effect of probiotics and CIK cells on regulating cell survival and proliferation signaling in liver tumors by Day 28 after CRC tumor induction

**Figure 9:**
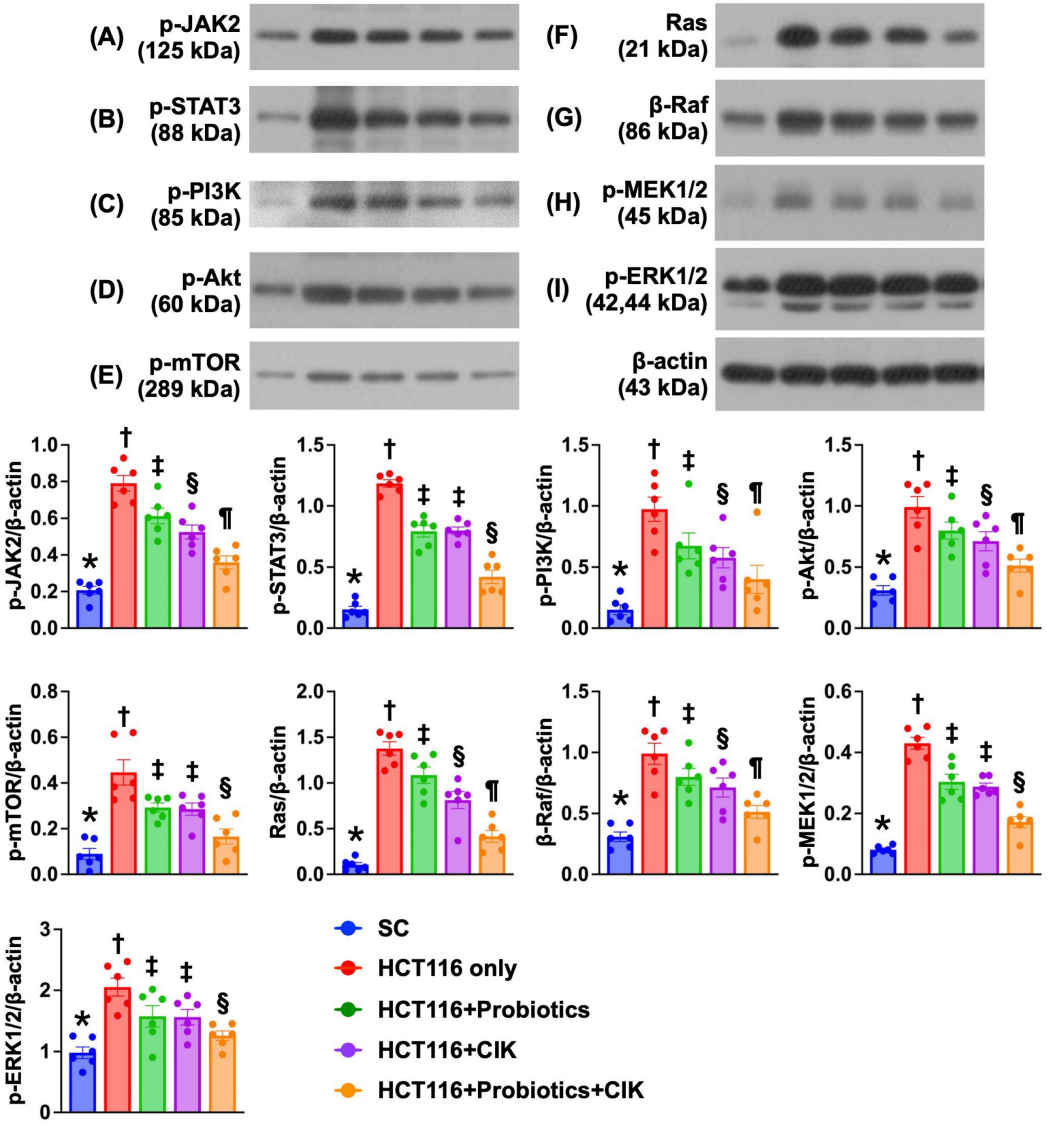
** Impact of probiotics and CIK on the regulating the cell survival and proliferation signalings in liver tumor tissue by day 28 after CRC tumor induction. A)** Protein expression of phosphorylated (p)-JAK2, * vs. other groups with different symbols (†, ‡, §, ¶) p<0.0001. **B)** Protein expression of p-STAT3, * vs. other groups with different symbols (†, ‡, §) p<0.0001. **C)** Protein expression of p-PI3K, * vs. other groups with different symbols (†, ‡, §, ¶) p<0.0001. **D)** Protein expression of p-Akt, * vs. other groups with different symbols (†, ‡, §) p<0.0001. **E)** Protein expression of p-mTOR, * vs. other groups with different symbols (†, ‡, §, ¶) p<0.0001. **F)** Protein expression of Ras, * vs. other groups with different symbols (†, ‡, §, ¶) p<0.0001. **G)** Protein expression of β-Raf, * vs. other groups with different symbols (†, ‡, §, ¶) p<0.0001. **H)** Protein expression of p-MEK1/2, * vs. other groups with different symbols (†, ‡, §) p<0.0001. **I)** Protein expression of p-ERK1/2, * vs. other groups with different symbols (†, ‡, §, ¶) p<0.0001. n = 6 for each group. CIK = cytokine-induced killer; CRC = colorectal cancer.

The protein expression levels of p-JAK and p-STAT1, i.e., components of the JAK-STAT1 signal transduction pathway, which plays an essential role in cell survival, proliferation and tumor formation, were lowest in Group 1 and significantly and progressively decreased from Groups 2 to 5 (Fig. 9 A-B). Additionally, the protein expression levels of p-PI3K, p-Akt and p-mTOR, three indices of cell stress/cell survival signaling biomarkers, exhibited identical patterns to that of the JAK-STAT1 signaling pathway biomarkers among the groups (Fig. 9 C-E).

Furthermore, the Ras/Raf/MEK/ERK signaling pathway plays crucial roles in the proliferation, survival and metastasis of CRC cells. Therefore, we also measured the protein levels of these signaling parameters, and the results revealed that the protein expression levels of Ras, β-Raf, p-MEK1/2 and p-ERK1/2, four biomarkers of cell proliferation/metastasis signaling, exhibited identical patterns to those of the JAK-STAT1 signaling pathway biomarkers among the groups (Fig. 9 F-I). In this way, our findings proved that these three types of cancer cell survival signaling pathways were activated in the liver parenchyma by Day 28 after CRC induction. Importantly, these signaling pathways were significantly suppressed by probiotics and CIK cells and further suppressed by the combined probiotics and CIK cell treatment.

## Discussion

This study, which included *in vitro* and *in vivo* investigations, provided several promising preclinical implications. First, the *in vitro* study demonstrated that the combination of probiotics and a high dose of CIK cells was superior to treatment with either one alone in suppressing CRC cell viability, colony formation, proliferation, metastasis, and biological activity. Second, probiotics and CIK cells had comparable activities in attenuating biomarkers of CRC adaptive immune resistance and signaling pathways for mediating biological and survival functions of CRC cells. Third, the *in vivo* experiments revealed that CRC activity and metastasis capacity were markedly suppressed by probiotics or CIK cell therapy and further suppressed by the combination of these two therapeutic regimens. Finally, CRC survival and proliferation signals were substantially inhibited by probiotics or CIK cell therapy and were further inhibited by the combination of these two therapeutic regimens, resulting in increased CRC cell apoptosis and death.

CRC metastasis is still an essential cause of highly unacceptable cancer-related mortality with a very uncommon long-term survival rate, indicating that the treatment of advanced CRC remains a formidable challenge for physicians, which encourages the development of a sufficiently innovative strategic management for patients with advanced CRC. Interestingly, increasing evidence 53, 54 has shown that probiotics play pivotal roles in preventing and inhibiting CRC through several possible mechanisms 53, including (1) changes in the intestinal microflora, (2) the suppression of carcinogenic compounds, (3) competition with pathogenic and putrefying microbiota, (4) the upregulation of the host immune response, (5) the inhibition of the differentiation and proliferative activity of cancer cells through the regulation of apoptosis, and (6) the inhibition of tyrosine kinase signaling pathways. Additionally, many previous investigations have shown that the transfusion of *ex vivo* expanded CIK cells effectively eliminates cancer cells 17-20. An essential finding of the present *in vitro* study was that the cell viability, growth, proliferation, and migration and invasive abilities of CRC cell lines (i.e., HCT 116 and SW620) were markedly suppressed by probiotics or CIK cells, which were also found to significantly and progressively suppress these abovementioned parameters in an increased dose-dependent manner. Importantly, the combination of probiotics and CIK cells offered greater benefits in suppressing the aforementioned parameters in CRC cell lines. Furthermore, *in vitro* studies revealed that these different regimens applied to CRC cell lines effectively augmented cellular apoptosis and mitochondrial damage, resulting in CRC death. In this way, the results of our *in vitro* studies, in addition to being consistent with the findings of previous studies 17-20, 26, 53, strengthened the merit of the innovative approach of combined probiotics and CIK cell therapy for CRC and therefore encouraged our team to conduct an animal model study to test the effect of this combined regimen on suppressing the abdominal and liver metastasis of CRC cells.

One of the important findings of the *in vivo* study was that by the end of the study period, the IVIS demonstrated that, compared with that in CRC without treatment, the bioluminescence signal, an indicator of tumor growth, was markedly reduced in animals with CRC by probiotics or CIK cells and further markedly reduced by the combined probiotics + CIK cell treatment. Additionally, the total number of harvested tumors, the tumor weights and amounts of ascites were markedly greater in animals with CRC without treatment, but these parameters were notably reduced in animals with CRC treated with probiotics or CIK cells and were further reduced with the combined probiotics + CIK cell regimens. Intriguingly, some clinical trials have shown that CIK cells combined with chemotherapy offer promising benefits compared with chemotherapy alone in patients with advanced lung cancer 22-26. In this way, the promising results of our *in vivo* study, in addition to strengthening the findings from previous studies 22-26, highlight that this combined regimen may be innovative for patients with advanced CRC, especially those refractories to conventional therapy.

The PD-L1 gene plays a fundamental role in cancer cell immune escape (i.e., adaptive immune resistance), and FAK plays an essential role in transmitting signals related to tumor cell proliferation, migration, adhesion and survival. Interestingly, our previous study revealed that FAK could also inhibit the ability of CIK cells to kill breast cancer cells 46, 47. A principal finding in the present study was that the protein levels of PD-L1 and FAK in harvested liver tumors were substantially suppressed by probiotics or CIK cells and further substantially suppressed by a combined probiotic and CIK cell treatment. On the other hand, the protein levels of apoptosis and DNA damage biomarkers were substantially increased in harvested liver tumors subjected to probiotic or CIK cell treatments and further increased in those subjected to combined therapy with probiotics and CIK cells. Accordingly, our findings, in addition to supporting the findings of our previous study, could, at least in part, explain why tumor eradication significantly and progressively increased, in order, by treatments with probiotics, CIK cells, and the combination of these two regimens.

JAK-STAT1, PI3K/Akt/mTOR and Ras/Raf/MEK/ERK, which are three cardinal signaling pathways, play similar crucial biological roles in cell survival, proliferation and tumor formation, growth and metastasis. Accordingly, it is necessary to investigate which signaling pathway plays a fundamental role in CRC growth, metastasis and survival. Thus, these three signaling pathways were thoroughly investigated. Surprisingly, we discovered that these three signaling pathways were significantly upregulated in CRC. However, these three signaling pathways were significantly suppressed by probiotics or CIK cells and further significantly suppressed by the combined therapy of probiotics + CIK cells. Accordingly, this *in vivo* study verified that these three signaling pathways together participate in CRC growth, proliferation, metastasis and survival. A distinctive finding was that these three signaling pathways were significantly suppressed by probiotics or CIK cell treatment and further significantly suppressed by the combination of probiotics and CIK cells. In this way, the results of the present *in vivo* study highlighted that perhaps combined regimens with probiotics + CIK cells could be an innovative therapy for patients with advanced CRC, especially those who are refractory to conventional therapy.

### Study limitations

**Figure 10:**
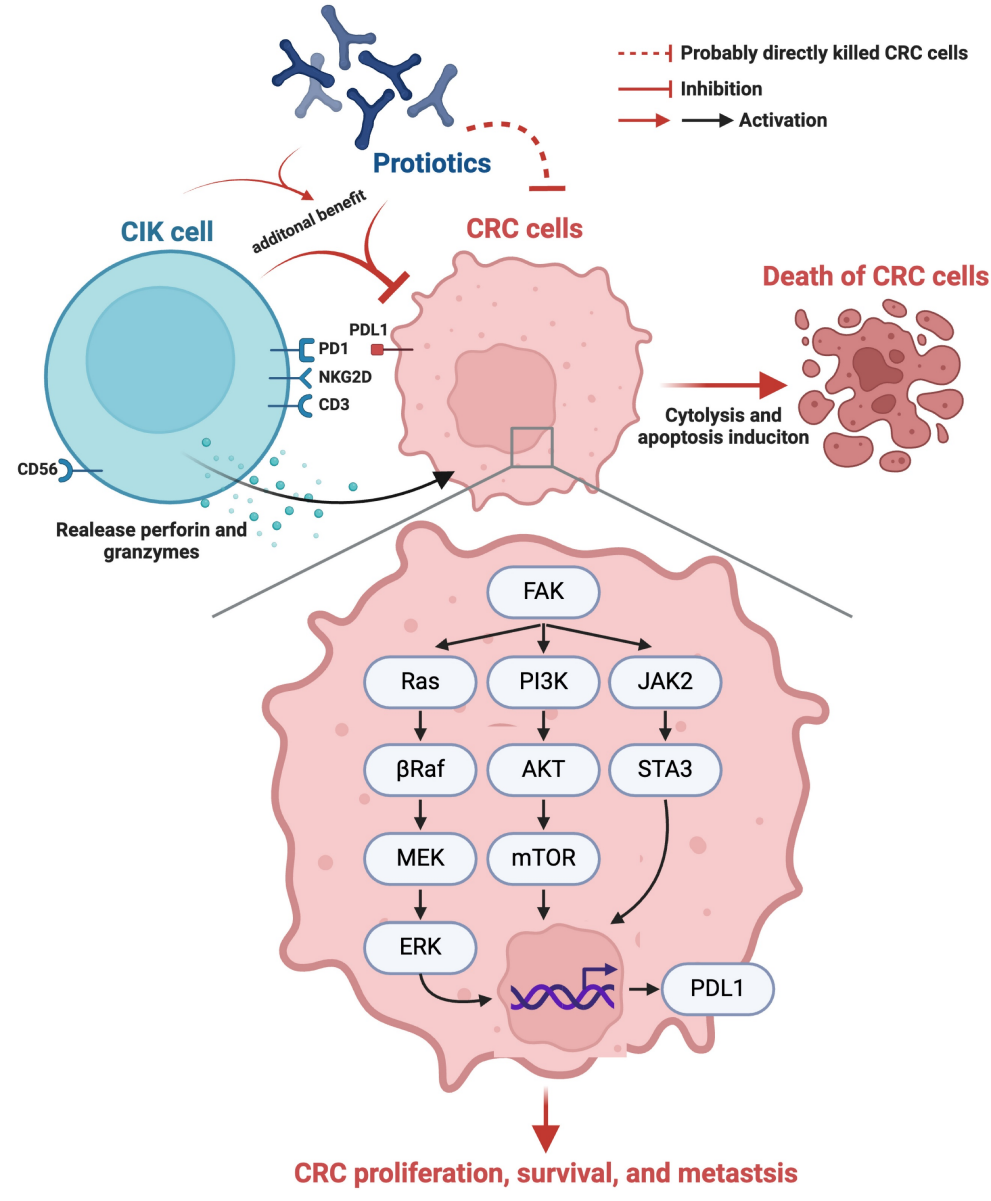
** Schematically illustrated the underlying mechanism of probiotics-CIK therapy on suppressing the pathological and biologic activities of CRC.** CIK = cytokine-induced killer; CRC = colorectal cancer.

Our *in vivo* study had several limitations. First, although the results were attractive and promising, the study period of 28 days was relatively short. Thus, the long-term outcome of the combined therapy with these two agents for CRC is still uncertain. Second, although extensive work was performed a total of three signaling pathways were studied, this study did not determine how many signaling pathways participate in CRC growth, proliferation, invasion and metastasis. On the basis of our *in vitro* and *in vivo* studies, we illustrate the underlying mechanism by which probiotic-CIK cell therapy suppresses the pathological and biological activities of CRC in Figure 10. Third, a fly in the ointment was that the *in vitro* study did not conduct the Western blot analysis for identification of cytosolic and mitochondrial cytochrome C levels because only immunofluorescent stain for identification of the lower expression of cytochrome C in mitochondria was not sufficient evidence of impaired mitochondrial integrity.

## Conclusions

In conclusion, the present study demonstrated that the implantation of CRC cells into the peritoneum of nude mice induced the dissemination of these CRC cells into the peritoneal cavity and their metastasis into liver, resulting in peritoneal carcinomatosis. Biomarkers of three fundamental cancer cell proliferation and survival signaling pathways and cancer cell immune escape/metastasis were shown to be upregulated in liver metastatic tumors. Probiotic-CIK cell therapy significantly suppressed these molecular-cellular perturbations, peritoneal carcinomatosis, and tumor growth.

## Abbreviations

CRC: colorectal cancer
CIK cells: cytokine-induced killer cells
IFN-γ: interferon-γ
IL-2: interleukin-2
PBMCs: peripheral blood mononuclear cells
FAK: focal adhesion kinase
PD-L1: programmed death-ligand 1
